# Supplementary material for: IgSF9b regulates anxiety behaviors through effects on centromedial amygdala inhibitory synapses
Source: Nat Commun. 2018 Dec 20;9:5400. doi: 10.1038/s41467-018-07762-1 (PMC6302093; doi:10.1038/s41467-018-07762-1)
Supplement: Supplementary file 2 — Supplementary Information [file 41467_2018_7762_MOESM2_ESM.pdf]

SUPPLEMENTARY INFORMATION

IgSF9b regulates anxiety behaviors through effects on centromedial amygdala inhibitory synapses

Babaev et al.

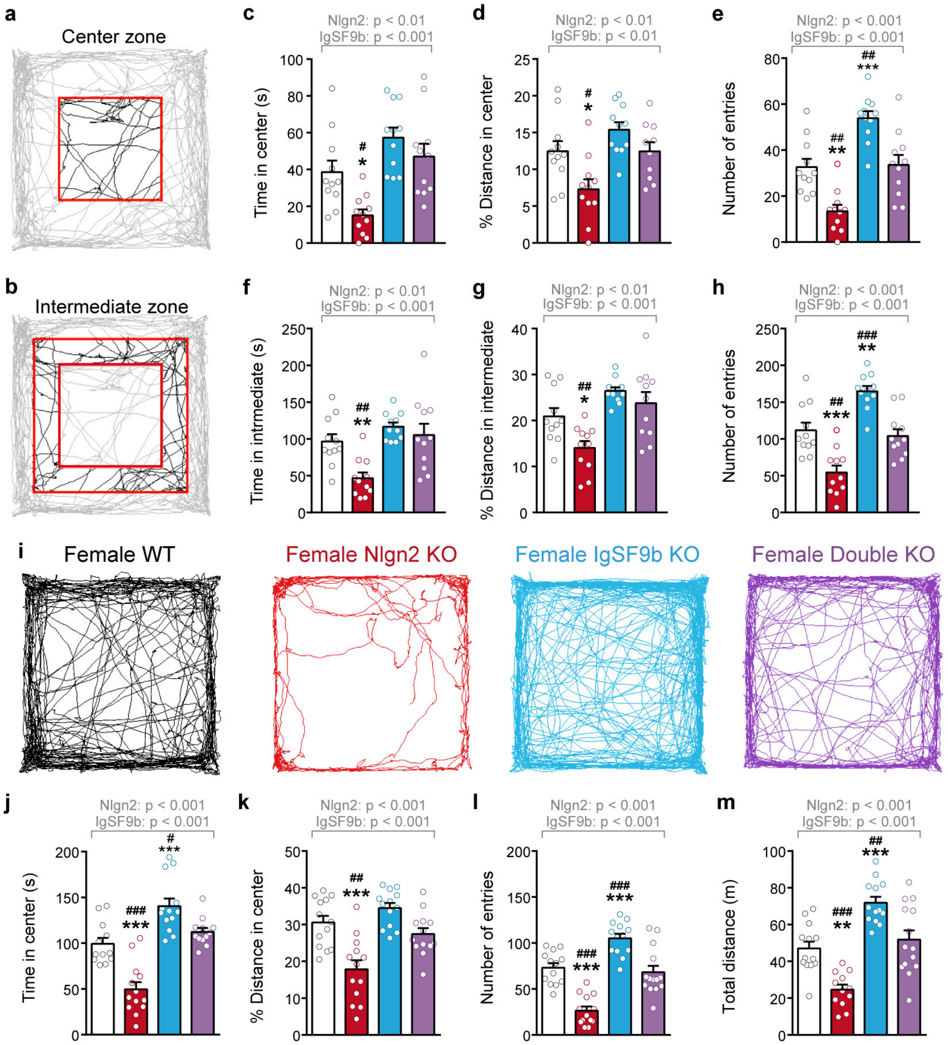

Supplementary Figure 1. Open field test results obtained from center and intermediate zone of open field chamber in male and female mice. a-b, Center and intermediate zone of the OF chamber (red squares). **c**, Time spent in the center of the OF. **d,** Distance traveled in the center of the OF, expressed as percentage of total distance traveled. **e**, Number of entries into the center of the OF. **f**, Time spent in the intermediate zone of the OF. **g**, Distance traveled in the intermediate zone of the OF, expressed as percentage of total distance traveled. **h**, Number of entries into intermediate zone of the OF. **i**, Representative traces of OF exploration by female WT, Nlgn2 KO, IgSF9b KO and double KO mice. **j**, Time spent by female mice in the anxiogenic center of the OF. **k**, Distance traveled by female mice in the anxiogenic center of the OF, expressed as percentage of total distance traveled. **l,** Number of entries by female mice into the center of the OF. **m**, Total distance traveled by female mice in the OF. Statistically significant ANOVA comparisons are marked in grey at the top of the panels and are listed in Supplementary Table 1. For all other ANOVA comparisons F < 1. Error bars represent SEM, n = 10-12 mice per genotype. WT, white bars; Nlgn2 KO, red bars; IgSF9b KO, blue bars; double KO, purple bars.

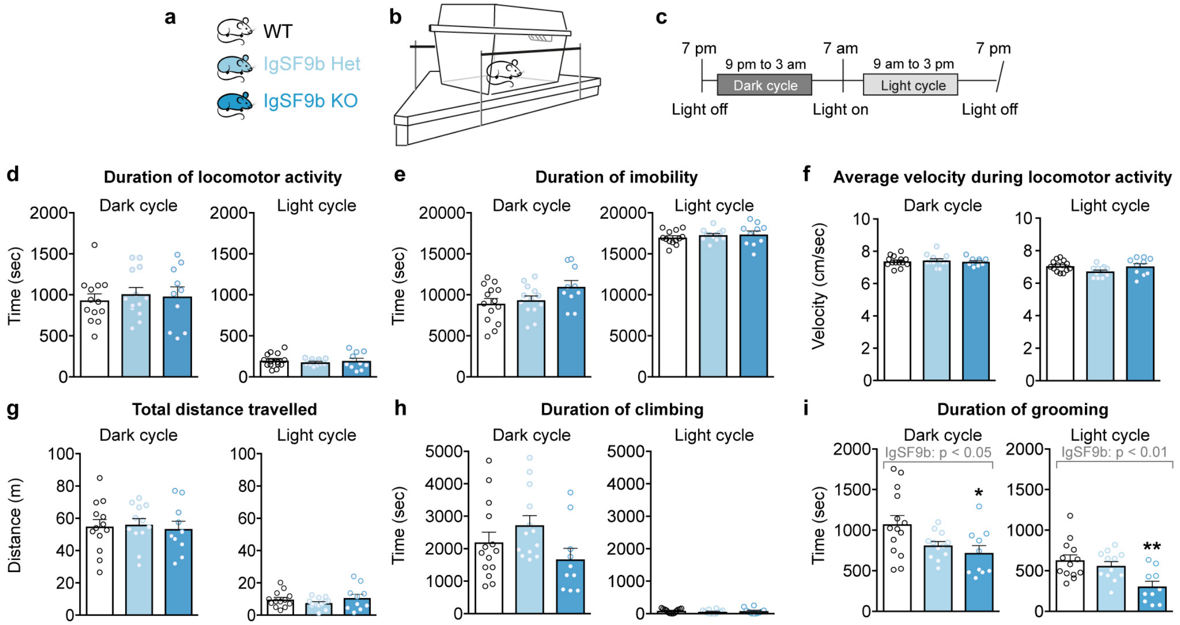

Supplementary Figure 2. Activity of IgSF9b Het and IgSF9b KO mice in home cage setting. a, WT and IgSF9b heterozygous and homozygous KO mice were tested for locomotor activity in a home cage setting. **b**, Diagram of the Laboras apparatus used for home cage testing. **c**, Diagram of the time periods of home cage activity recording. Data were recorded for 6 hours each during the dark cycle and the light cycle. **d**, Duration of locomotor activity; **e**, Duration of immobility; **f**, Average velocity during locomotor activity; **g**, Total distance travelled; **h**, Duration of climbing; **i**, Duration of grooming of IgSF9b KO, IgSF9b Het, and WT mice during dark and light cycle. One-way ANOVA for duration of grooming: $F_{2,33} = 4.29$, $p < 0.05$ (dark cycle); $F_{2,32} = 7.33$, $p < 0.01$ (light cycle). For all other ANOVA comparisons $F < 1$. All bars represent mean + SEM, * $p < 0.05$, ** $p < 0.01$. n = 14-18 mice per genotype.

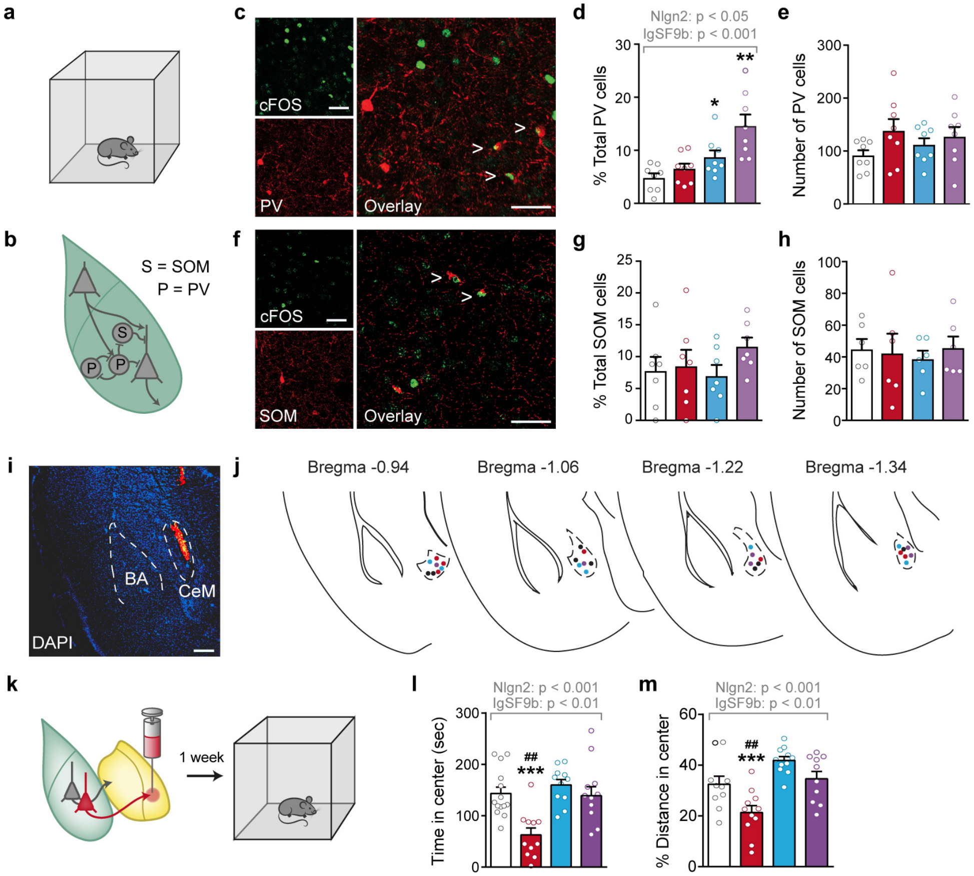

Supplementary Figure 3. cFos colocalization with PV and SOM-positive interneurons and with Retrobeads in basal amygdala. a, Experimental design of cFos induction assay: mice of all four genotypes were exposed to the OF for 10 min, and 90 min later they were perfused for cFos and PV/SOM immunolabeling. b, Diagram of local inhibitory circuit in basal amygdala. **c**, Representative images of cFos and PV immunolabeling in BA. Scale bar, 50 µm. **d**, Percentage of cFos-positive PV interneurons out of total number of PV interneurons. **e,** Total number of PV interneurons. **f,** Representative images of cFos and SOM immunolabeling in BA. Scale bar, 50 µm. **g**, Percentage of cFos-positive SOM interneurons out of total number of SOM interneurons. **h**, Total number of SOM interneurons. **i**, Representative photomicrograph of site of injection of Retrobeads (red) and dapi staining (blue) in CeM. Scale bar, 200 µm. **j**, Sites of injection of Retrobeads. Black dots = WT mice, red dots = Nlgn2 KO mice, blue dots = IgSF9b KO mice, purple dots = double KO mice. **k**, OF test was conducted one week after the injection of the Retrobeads. **l**, Time spent in the anxiogenic center of the OF during cFos induction following Retrobead injection. **m**, Distance traveled in the center of the OF during cFos induction following Retrobead injection, expressed as percentage of total distance traveled. Statistically significant ANOVA comparisons are marked in grey at the top of the panels and are listed in Supplementary Table 1. For all other ANOVA comparisons, F < 1. All bars represent mean + SEM. * $p < 0.05$, ** $p < 0.01$, *** $p < 0.01$ compared to WT; $^{\#\#}$ $p < 0.01$ compared to double KO. n = 7-8 mice per genotype for the double-labeling of cFos with PV and SOM, and n = 11-14 mice per genotype for the retrograde tracing experiment. WT, white bars; Nlgn2 KO, red bars; IgSF9b KO, blue bars; double KO, purple bars.

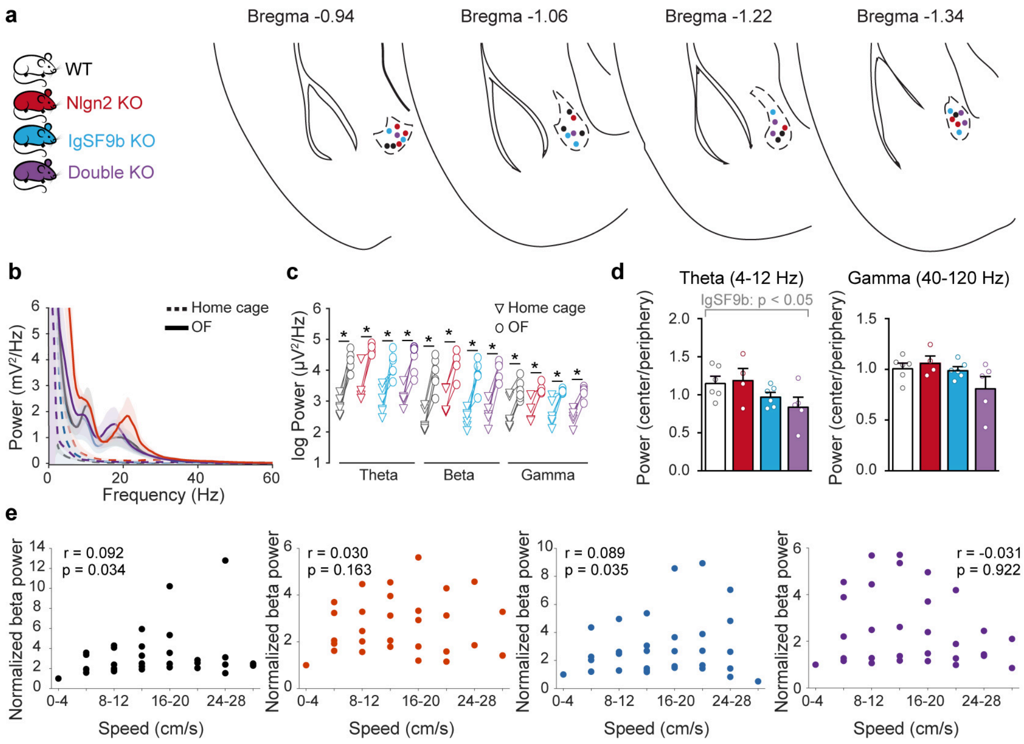

Supplementary Figure 4. Electrode placement and analysis of LFP power in WT, Nlgn2 KO, IgSF9b KO and double KO mice. a, Electrode placements in CeM for all animals used in analysis. **b,** LFP power spectrum for all four genotypes during home cage activity and exploration of the entire OF. **c,** CeM power quantification between home cage and OF for different frequency bands. **d,** Average power increase in the center relative to the periphery of the OF for the theta (4-12 Hz) and gamma (40-120 Hz) frequency range. Theta: Two-way ANOVA, $F_{1,17} = 6.95$, $p < 0.05$ (main effect of IgSF9b). **e,** Changes in normalized beta power as a function of speed during exploration of the OF. For all other ANOVA comparisons, $F < 1$. Error bars represent SEM. * $p < 0.05$, Wilcoxon rank sum test. n = 4-6 mice per genotype. WT, white bars; Nlgn2 KO, red bars; IgSF9b KO, blue bars; double KO, purple bars.

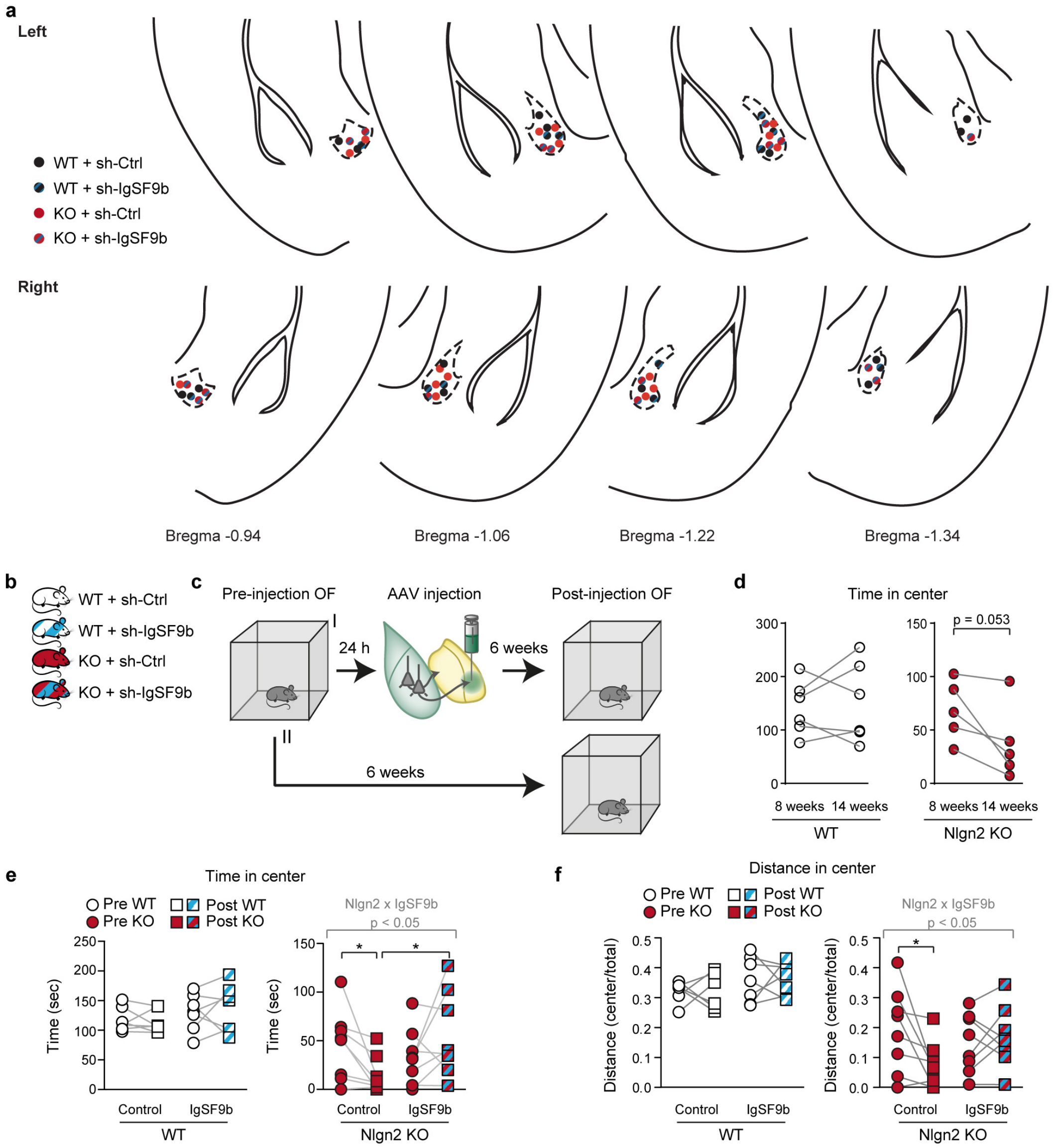

Supplementary Figure 5. Bilateral injection sites of AAV-shRNA particles and effects of local knockdown of IgSF9b in the adult CeM. **a,** Virus injection sites for all animals used in analysis. Each dot corresponds to the brightest GFP fluorescence in the CeM of AAV injected mice. Only mice in which most of the GFP expression was restricted to the borders of CeM were included in the behavioral analysis. Anteroposterior stereotaxic coordinates relative to Bregma are indicated below the schematics. **b**, Representation of experimental groups generated. **c**, Schematic diagram showing experimental design of IgSF9b-shRNA experiment including the measurement of age effect on the anxiety phenotype of WT and Nlgn2 KO mice. **d**, Time in center in 8 week old and 14 week old WT (left panel) and Nlgn2 KO (right panel) mice. **e**, Time in center pre- and post-injection in WT (left panel) and Nlgn2 KO (right panel) mice. WT: Two-way ANOVA, $F_{1,11} = 1.36$, $p = 0.268$ (main effect of shRNA). KO: Two-way ANOVA, $F1_{,14} = 1.19$, $p = 0.294$ (main effect of shRNA), $F_{1,14} = 7.29$, $p = 0.017$ (interaction between time point of OF and shRNA). **f**, Normalized distance in center pre- and post-injection in WT (left panel) and Nlgn2 KO (right panel) mice. WT: Two-way ANOVA, $F_{1,11} = 3.61$, $p = 0.084$ (main effect of shRNA). KO: Two-way ANOVA, $F_{1,14} = 2.60$, $p = 0.129$ (time point of the OF, i.e. pre vs post surgery); $F_{1,14} = 5.44$, $p = 0.035$ (interaction between time point of OF and shRNA). For all other ANOVA comparisons $F < 1$. Posthoc analysis: * $p < 0.05$ relative to WT, ** $p < 0.01$ relative to WT. Error bars represent SEM, n = 6-9 mice per group.

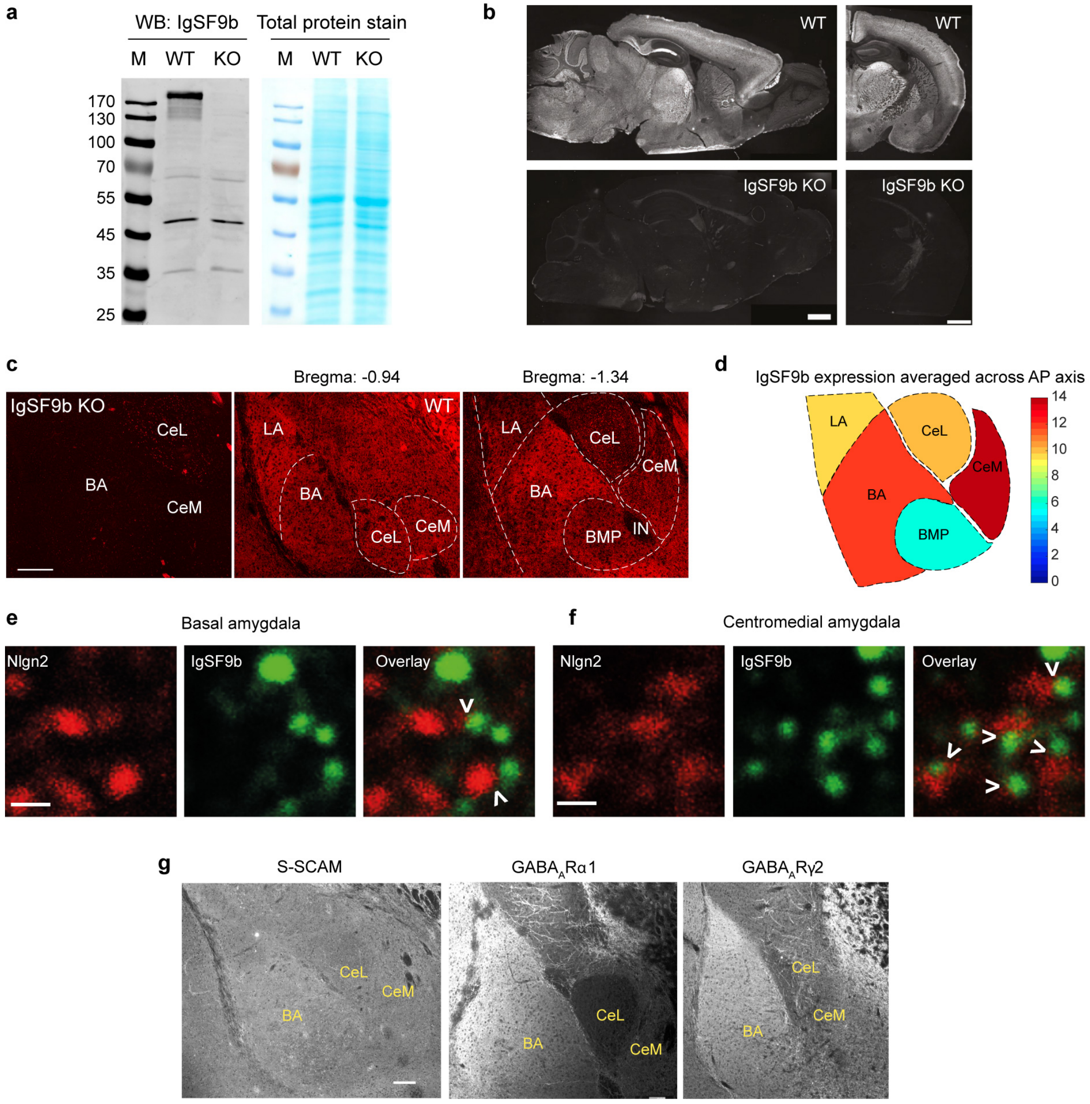

Supplementary Figure 6. IgSF9b expression pattern and co-localization of IgSF9b and Nlgn2 in BA and CeM. a, Immunoblot to assess specificity of the IgSF9b antibody using whole-brain tissue from WT and IgSF9b KO mice. Numbers next to the protein ladder represent molecular weight in kDa. Equal loading was confirmed using the Pierce reversible protein stain kit (ThermoFisher Scientific). **b,** Photomicrographs of immunostaining for IgSF9b in sagittal and coronal sections from WT and IgSF9b KO mice. Scale bars, 1 mm. **c,** Low magnification overview of IgSF9b expression in amygdala of IgSF9b KO (left panel) and WT mouse (middle panel-anterior part; and right panel-posterior part of amygdala). Anteroposterior stereotaxic coordinates relative to Bregma are indicated above the panels, Scale bar, 200 μm. **d,** Summary diagram of the average fluorescence intensity in various regions of amygdala in mice immunolabeled with IgSF9b antibody. In total, fluorescence from 6-10 high magnification photomicrographs taken across AP axis (Bregma: -0.94 to -1.34) from each area in each out of two WT mice were measured and the fluorescence intensity of matching anatomical region in KO mouse was subtracted prior to averaging to calculate the final intensity value for each brain region. BMP: posterior basal amygdala (magnocellular division). **e-f,** Photomicrographs of Nlgn2 and IgSF9b puncta in BA (e) and CeM (f). Scale bar, 500 nm. **g,** Photomicrographs of immunostaining for S-SCAM, GABA$_A$Rα1 and GABA$_A$Rγ2 in BA, CeM and CeL. Scale bar, 200 μm.

Supplementary Table 1. Two-way ANOVA comparisons for supplementary figures.

Figure	Main effect of Nlgn2 KO		Main effect of IgSF9b KO		Nlgn2 x IgSF9b interaction	
	F-value	p-value	F-value	p-value	F-value	p-value
**Sup 1c**	$F_{1,40} = 8.85$	0.005	$F_{1,40} = 19.92$	0.0001	$F_{1,40} = 1.38$	0.247
**Sup 1d**	$F_{1,39} = 10.35$	0.003	$F_{1,39} = 10.28$	0.003	$F_{1,39} < 1$	n.s.
**Sup 1e**	$F_{1,40} = 32.12$	0.0001	$F_{1,40} = 35.15$	0.0001	$F_{1,40} < 1$	n.s.
**Sup 1f**	$F_{1,40} = 8.77$	0.005	$F_{1,40} = 14.50$	0.0005	$F_{1,40} < 1$	n.s.
**Sup 1g**	$F_{1,40} = 7.57$	0.009	$F_{1,40} = 19.34$	0.0001	$F_{1,40} = 1.49$	0.230
**Sup 1h**	$F_{1,40} = 41.43$	< 0.0001	$F_{1,40} = 31.39$	< 0.0001	$F_{1,40} < 1$	n.s.
**Sup 1j**	$F_{1,46} = 30.47$	< 0.0001	$F_{1,46} = 54.27$	< 0.0001	$F_{1,46} = 2.33$	0.134
**Sup 1k**	$F_{1,48} = 27.88$	< 0.0001	$F_{1,48} = 13.02$	0.001	$F_{1,48} = 2.31$	0.135
**Sup 1l**	$F_{1,49} = 58.73$	< 0.0001	$F_{1,49} = 45.66$	< 0.0001	$F_{1,49} < 1$	n.s.
**Sup 1m**	$F_{1,48} = 29.87$	< 0.0001	$F_{1,48} = 44.78$	< 0.0001	$F_{1,48} < 1$	n.s.
**Sup 3d**	$F_{1,28} = 7.17$	0.012	$F_{1,28} = 17.66$	< 0.001	$F_{1,28} = 2.14$	0.155
**Sup 3e**	$F_{1,28} = 3.50$	0.072	$F_{1,28} < 1$	n.s.	$F_{1,28} < 1$	n.s.
**Sup 3g**	$F_{1,20} = 1.71$	0.204	$F_{1,20} < 1$	n.s.	$F_{1,20} < 1$	n.s.
**Sup 3l**	$F_{1,44} = 13.16$	< 0.001	$F_{1,44} = 11.04$	0.002	$F_{1,44} = 4.56$	0.038
**Sup 3m**	$F_{1,44} = 14.74$	< 0.001	$F_{1,44} = 11.71$	0.001	$F_{1,44} = 3.64$	0.063
**Sup 4d (theta)**	$F_{1,17} < 1$	n.s.	$F_{1,17} = 6.95$	< 0.05	$F_{1,17} < 1$	n.s.
